# Prior corticosteroid treatment alters cPBMC composition and IFNγ response to immunotherapy in canine cancer

**DOI:** 10.3389/fimmu.2025.1544949

**Published:** 2025-04-24

**Authors:** Anna Barbara Emilia Zimmermann, Betül Taskoparan, Daniel Fuchs, Stanislav Pantelyushin, Mathischan Maheswaran, Manuela Schnyder, Sonja Hartnack, Carla Rohrer Bley, Johannes vom Berg

**Affiliations:** ^1^ Clinic for Radiation Oncology and Medical Oncology, University Animal Hospital, Vetsuisse Faculty, University of Zurich, Zurich, Switzerland; ^2^ Institute of Laboratory Animal Science, Vetsuisse Faculty, University of Zurich, Schlieren, Switzerland; ^3^ Institute of Veterinary Pharmacology and Toxicology, Vetsuisse Faculty, University of Zurich, Zurich, Switzerland; ^4^ Institute of Parasitology, Vetsuisse Faculty, University of Zurich, Zurich, Switzerland; ^5^ Section of Epidemiology, Vetsuisse Faculty, University of Zurich, Zurich, Switzerland

**Keywords:** immunotherapy, immune checkpoint inhibitor, cancer, atezolizumab, gilvetmab, corticosteroid, IL-12, prednisolon

## Abstract

**Background:**

Immunotherapy using immune checkpoint inhibitors (ICIs) represents a promising therapeutic approach for canine cancer patients. Similar to human cancer patients, the concurrent use of corticosteroids may attenuate the efficacy of immune checkpoint inhibitors in dogs. In this study, we evaluated the impact of corticosteroid therapy on canine peripheral blood mononuclear cell (cPBMC) composition and the in vitro response to Programmed Death-1/Programmed Death-Ligand 1 (PD-1/PD-L1) axis blockade and recombinant human Interleukin-12 (rhIL-12) stimulation.

**Methods:**

cPBMC samples were collected from 24 healthy, 44 cancer-bearing untreated, and 33 cancer-bearing corticosteroid pre-treated dogs. Lymphocytes were polyclonally stimulated with Staphylococcal Enterotoxin B (SEB) and either atezolizumab, a cross-functional anti-PD-L1 ICI, or rhIL-12. We analyzed the absolute and relative changes in canine interferon-gamma (cIFNɣ) production. Stimulation with gilvetmab, a recently developed canine anti-PD-1 ICI, revealed comparable results to atezolizumab. Moreover, we assessed the influence of corticosteroid pre-treatment on cPBMC composition by flow cytometry.

**Results:**

Corticosteroid treatment significantly affected the immune profile, primarily the monocytic compartment, and functional cIFNɣ response of cPBMCs. Nevertheless, responses to immunotherapy appeared to be highly individual.

**Conclusions:**

Overall, we observed trends suggesting that prior corticosteroid therapy may compromise the efficacy of PD-1/PD-L1 axis blockade and IL-12 in dogs with cancer. While the dose and timing of corticosteroid administration in this study reflected clinical reality and would not justify withholding this emerging therapeutic option, corticosteroid pretreatment may be a confounder for PD-1/PD-L1 axis blockade or IL-12 therapy in canine oncology.

## Introduction

Immune checkpoints are part of the normal immune system and are responsible for controlling immune responses. PD-1 (Programmed Death-1), expressed predominantly on T cells upon antigen encounter, and PD-L1 (Programmed Death-Ligand 1), found on antigen-presenting and cancer cells, are key checkpoint molecules that negatively regulate anti-tumor immunity (1). Tumors evade the immune system by activating immune checkpoint pathways, neutralizing anti-tumor immune responses. Immune checkpoint inhibitors (ICIs) counter this by relieving T cell suppression, promoting immune activation in both tumor and peripheral tissues to attack cancer (1, 2). The PD-1/PD-L1 pathway is currently the most commonly targeted checkpoint in human oncology. Another approach to reactivate the immune system employs pro-inflammatory cytokines such as interleukin-12 (IL-12), which activates NK cells, effector CD4 T cells and cytotoxic CD8 T cells and induces IFNγ expression for direct tumor cell targeting and antiangiogenic effects (3). In canine models, human IL-12 is cross-functional and has shown anti-cancer activity by stimulating cIFNγ production, promoting anti-tumor immune responses (4–7). Pro-inflammatory cytokines such as IL-2, IL-15 and IL-12 may enhance therapeutic outcomes of ICI treatment (8).

Companion dogs spontaneously develop cancers with molecular and biological similarities to human tumors (9–11). While ICIs are approved for an increasing number of human cancers, they do not show efficacy in all patients, posing a substantial challenge to their general application (12). Similar to human studies, ICI and IL-12 have triggered treatment responses in canine cancer patients (13–15). Therefore dogs, due to similar tumor-host immune interactions, offer a valuable model to study ICI-corticosteroid interactions (11, 16). During cancer treatment, dogs often receive corticosteroids, which, while helpful in some cancer types or relieving tumor-associated symptoms (17), may also suppress CD4 and CD8 T cell activation and induce leukopenia, potentially diminishing the efficacy of ICI therapy (18). These effects appear dose-dependent and diminish over time (19), nevertheless, no clear cut-off value has been identified for ICI therapy. In addition, systematic reviews in human medicine reached different conclusions on the interaction between ICI and corticosteroids: one systematic review found no significant difference in progression-free and overall survival time with or without corticosteroid use (20), while another identified corticosteroid use as an unfavorable prognostic factor in ICI-treated cancer patients (21). To better understand which cancer patients might benefit from ICI treatment, the role of corticosteroid use requires further investigation.

Gilvetmab, a dog-specific PD-1 ICI, was recently approved in the US (22) but remained unavailable for general practice outside North America. In light of the limited availability of veterinary ICIs, human anti-PD-L1 ICI atezolizumab has shown cross-reactivity and functionality with canine cells, thus representing a convenient research reagent (23). Here, we investigated the effect of prior treatment with corticosteroids on peripheral lymphocytes and on the *ex vivo* response to both checkpoint blockade or IL-12 as a potential future combination partner (4, 24). First, we examined the effect of corticosteroids on peripheral blood mononuclear cells (PBMC) derived from tumor-bearing dogs, comparing cIFNγ production between corticosteroid-treated and untreated dogs. Additionally, flow cytometry was used to assess the PBMC composition in relation to corticosteroid administration, comparing cell-type percentages across healthy, tumor-bearing, corticosteroid-treated and tumor-bearing, corticosteroid non-treated dogs.

## Materials and methods

The current investigation involved analysis of PBMCs derived from healthy and tumor-bearing dogs treated with or without corticosteroids regarding their composition and reaction to stimulation with immune therapeutics. Atezolizumab was used as the ICI for this study based on systematic testing of approved human checkpoint inhibitors for cross-reactivity and cross-functionality with canine lymphocytes (23). Durvalumab, an anti-PD-L1 ICI, which shows no specific binding to canine cells was used as a control (23). rhIL-12 is known for its anti-cancer activity and ability to trigger the production of cIFNγ in previous canine clinical studies (4, 5). A previously established 14-color canine-specific flow cytometry panel was used to assess the composition of the cPBMCs (25). The data was analyzed using R (2024) (26) (**Supplementary Figure 1**).

### Patient inclusion criteria and sampling methods

The tumor-bearing dogs in this study were privately owned patients presented to the Clinic for Radiation Oncology & Medical Oncology, University Animal Hospital, University of Zurich, Switzerland. Informed consent was acquired from all owners prior to enrolment. The healthy dogs for this study were blood donors at the University Animal Hospital. Blood samples were collected during routine medical procedures and sample collection did not present an additional constraint. All animals were assessed in acceptable general conditions for blood collection before each sample was taken. Dogs were grouped as healthy donors, tumor-bearing corticosteroid treated donors, and tumor-bearing non corticosteroid treated donors. Corticosteroids for treated donors were administered up to the day of sampling. When the corticosteroid treatment was being tempered down a mean dose was calculated over the whole period of administration. Each group consisted of dogs of different sexes, ages, and breeds to reach a heterogeneous distribution between the groups to best represent the cases seen in the clinic. It was not possible to randomize dogs for corticosteroid treatment because corticosteroids were administered in accordance with clinical indications. The project was examined and approved by the veterinary office for animal testing in Zurich (permit: ZH171/18). PBMCs from healthy beagles for functional testing of gilvetmab were collected under the cantonal veterinary office permit ZH242/17.

The inclusion criteria for tumor-bearing dogs were good overall health as checked by a veterinarian, no prior chemotherapy, radiation therapy, or tumor-removal surgery, more than 5 kg of weight, and routine bloodwork that did not contradict blood sampling. For identifying tumor types, biopsies were taken from the tumors if feasible. Otherwise, tumor types were classified by either an MRI or a CT imaging read by a board-certified radiologist. Healthy donors could be sampled multiple times if presented more than once for blood donation. Blood was drawn via venous puncture (V. cephalica/jugularis). For dogs under 10 kg 4ml of blood was drawn, and over 10 kg 8ml of blood was taken. Corticosteroids were administered according to “lege artis” and the decision was made by the veterinarian in charge. Corticosteroid treatment consisted of oral or rarely intravenous prednisolone.

### Isolation of canine peripheral blood mononuclear cells

Whole blood was collected from patients in EDTA anticoagulant-containing tubes. cPBMCs were isolated from the obtained blood samples in accordance with Pantelyushin et al. (23). The acquired cPBMCs were counted using cell counting chambers (Nexcelom, Massachusetts, USA) and resuspended in freezing medium (50% RPMI1640 (R8758-500ML, SIGMA-ALDRICH), 40% FBS SUPERIOR (SO615, SIGMA-ALDRICH), 10% DMSO (A3672,0050, PanReacAlliChem)) at 1x10^6^ cells/ml for storage in cryotubes (Techno Plastic Products AG, 89012 Trasadingen, Switzerland) in a freezing container at -80°C for 48h and then transferred to -150°C. For analysis, samples were thawed in a water bath at 37°C. The content of the aliquots was then washed with 10ml of complete medium (500ml RPMI1640 (R8758-500ML, SIGMA-ALDRICH), 50ml FBS SUPERIOR (SO615, SIGMA-ALDRICH), 5ml GlutaMAX (35050-038, gibco), 5ml Pen Strep Penicillin Streptomycin (15140-122, gibco), 5ml Sodium Pyruvate (11360-039, gibco), 5ml NEAA (11140-050, gibco), 12,5ml HEPES Buffer Solution (15630-056, gibco)). The supernatant was removed and only the cells were used for immediate downstream analysis via either flow cytometry or ELISA.

### Cultivation and *in vitro* treatment of cPBMCs

After washing, cPBMCs were recounted and diluted in complete medium to the concentration of 2×10^5^ cells/ml. Cell stimulation and activation were performed as previously described by Pantelyushin et al. (23). Briefly, cells were stimulated with 50 ng/ml Staphylococcal Enterotoxin B (SEB) (BT202, Toxin Technology, Sarasota, FL, USA) and 10 µg/ml atezolizumab or alternatively 10 µg/ml durvalumab (both obtained from H.L. at Department of Medical Oncology, Department of Internal Medicine, University Hospital Basel, Basel, Switzerland), or 50 ng/ml SEB and 1ng/ml rhIL-12 (Recombinant Human IL-12 p70 (HEK293 derived), Peprotech, London, UK). Similarly, cPBMCs from healthy beagles were stimulated with 50 ng/ml SEB and 10 µg/ml gilvetmab (MA5-42149, Thermo Fischer Scientific) or its isotype control (canine IgG2 isotype control antibody HyHEL-10, Proteogenix). When possible, each stimulation was carried out in biological triplicates. The treatment of cPBMCs was carried out on a sterile 96 well plate (Corning, 353077, New York, USA) with a final volume of 200 ml per well. The plate was incubated at 37°C for 72 hours. After incubation, the plate was centrifuged, and the supernatant was transferred to another 96 well plate and stored at -20°C.

### Measurement of cIFNγ production of treated cPBMCs

Each supernatant sample was analyzed as a technical duplicate. Concentrations of cIFNγ were measured using a canine-specific ELISA kit (3113-1H-6, Mabtech, Nacka Strand, Sweden) in accordance with manufacturer guidelines using half the volume intended. Samples were diluted 1:9 to conform to the detection limit and analyzed alongside a cIFNγ standard curve decreasing from 800 to 12.5 pg/ml. The absorbance levels were measured with a SPARK plate reader (30086376, TECAN, Männedorf, Switzerland).

### Determination of cPBMC composition with flow cytometry

Washed cPBMCs were transferred to a 96 well plate (CLS353077, Corning, New York, USA) for staining as previously described (25). First, cells were stained with a fixable live/dead cell staining. The surface antigen staining antibodies were mixed at their respective dilutions and were added to the cells and, as single staining, to beads for machine calibrations. Afterward, cells and beads were fixed with a 3:1 mixture of Cytofix and Cytoperm. At last, the intracellular staining was added as a mixture to each sample. Identical to the surface staining, beads were stained with each individual antibody as well for machine calibrations. The cells were acquired within 30 minutes after being resuspended with the BD LSR Fortessa II (BD Bioscience, San Jose, California, USA) (25). Gating and analyses were done using FlowJo software (V.10.7.1; BD Bioscience). Samples were stained and run as two batches within four consecutive days, with no changes in the machine settings or maintenance taking place in between. To control comparability between the two different flow cytometry runs, control samples were used, calibration beads were freshly stained for each run and all gates were set individually in accordance with previous findings (25) (**Supplementary Figure 2**).

### Statistical methods and tests

Clinical data was collected from the dogs’ records. The database included: sex, age, weight, breed, previous treatments such as surgery, radiation, or chemotherapy, tumor type, corticosteroid administration, duration, and dose. Where cPBMCs were stimulated in triplicates and ELISA measurements were taken in duplicates; a mean was calculated for each patient and each stimulation. Statistical calculations were conducted with R (R version 4.1.2) (26). The packages “plyr” (27), “dplyr” (28), and “tidyr” (29) were used for data curation and sorting. “DescTools” (30) was used to determine binomial or multinomial 95% confidence intervals using the Jeffreys method. Pie charts and stacked bar graphs were plotted for tumor-bearing and healthy dogs to gather information about influencing factors and distribution. Boxplots were generated to visualize differences in normalized cIFNγ production after stimulation between different groups and Mann-Whitney U test was performed between the groups. A p-value of <0.05 was considered significant. Dot plot was used to show the differences between stimulations on an individual base. A scatterplot was generated for tumor-bearing corticosteroid treated dogs to show the distribution of dose and duration of corticosteroid administration using “ggplot2” (31) and “ggbreak” (32). To determine whether there is a connection between corticosteroids given and cIFNγ output after stimulation of cPBMCs of tumor-bearing dogs a scatterplot was generated and a linear regression line was added to visualize trend development with “ggplot2” (31). R-squared was calculated for the trendlines to quantify the correlation between the variables. For obtaining predictive values to differentiate between tumor-bearing and healthy dogs while including corticosteroid treatment as a covariate, a logistic regression with all complete cases was undertaken and the predicted values further analyzed using a ROC curve to decide on diagnostic predictability (33). The results from the flow cytometry analysis were grouped and cPBMC compositions were compared by Kruskal-Wallis test using GraphPad Prism version 10.2.3 for Mac OSX (GraphPad Software, Boston, Massachusetts, USA). Data visualization and illustration were created and adjusted using R Studio, GraphPad Prism, and Adobe Illustrator 2025 version 29.0.1 (Adobe Inc., San Jose, CA).

## Results

### Study population

108 blood samples from 100 individual dogs were collected. Seven healthy blood donors were sampled twice. One dog with lipoma was sampled once and then resampled as he developed a sino-nasal tumor. 76 dogs were tumor-bearing, and 24 were healthy dogs presented for blood donations. As for testing with gilvetmab two female intact and three male intact, 2-year-old, healthy beagles were sampled.

**Figure 1:**
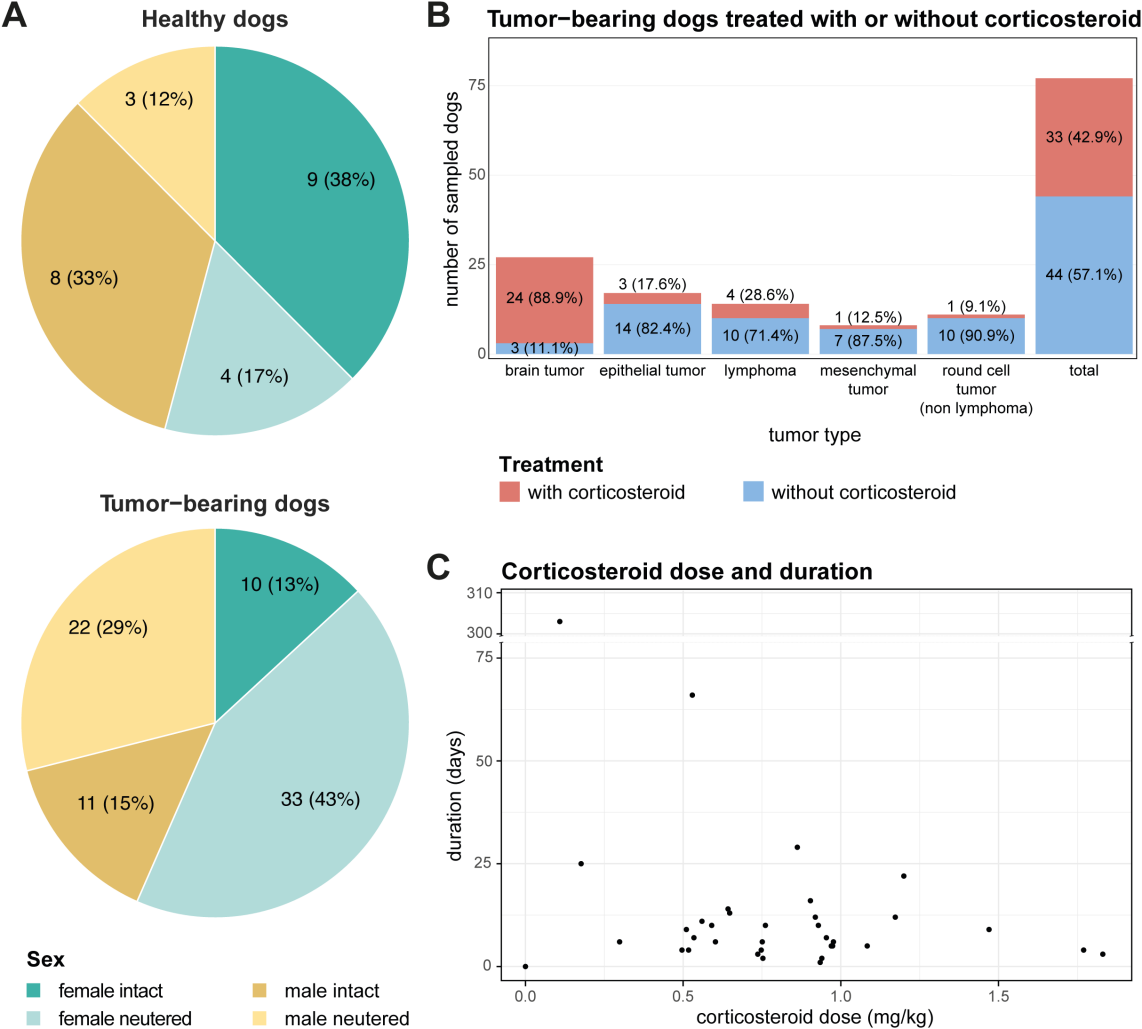
Distribution and characteristics of enrolled patients. **(A)** Pie charts illustrating the distribution of sex and neutering status among enrolled patients, categorized into healthy (top) and tumor-bearing dogs (bottom). **(B)** Distribution of tumor types among tumor-bearing patients separated by corticosteroid administration for each tumor type. **(C)** Scatter plot demonstrating the application of corticosteroid treatment among tumor-bearing patients, with the x-axis representing corticosteroid dose and the y-axis representing treatment duration in days.

More neutered female and male dogs were in the tumor-bearing group than in the healthy group (**Figure 1A**). The most represented breed among the tumor-bearing dogs were cross-breed dogs (**Supplementary Table 1**). Overall, the tumor-bearing dogs were older and lighter in comparison to the healthy dogs (**Supplementary Table 2**). 24 out of 27 dogs with a brain tumor received corticosteroids before being sampled due to neurological symptoms. In comparison, dogs with epithelial tumors were rarely treated with corticosteroids. As for the mesenchymal and round cell (non-lymphoma) bearing dogs, only one dog per group received corticosteroids before sampling (**Figure 1B**). The duration of corticosteroid treatment before sampling varied with a mean of 19.6 days (range 1-303 days). On average a corticosteroid dose of 0.8 mg/kg/day (range 0.1-1.8 mg/kg/day) was administered (**Figure 1C**). The highest dose was given to a dog with a mesenchymal tumor (1.5 mg/kg/day), and the lowest dose to a dog with lymphoma (0.1 mg/kg/day). The mean given dose per day varied strongly. Overall, the duration and dose of corticosteroids given show a broad distribution, which might represent the clinician’s choice more than adherence to a certain protocol for different diseases.

### Prior corticosteroid treatment dampens IFNγ production of dog PBMCs upon *ex vivo* stimulation with rhIL-12 or PD-1/PD-L1 blockade

**Figure 2:**
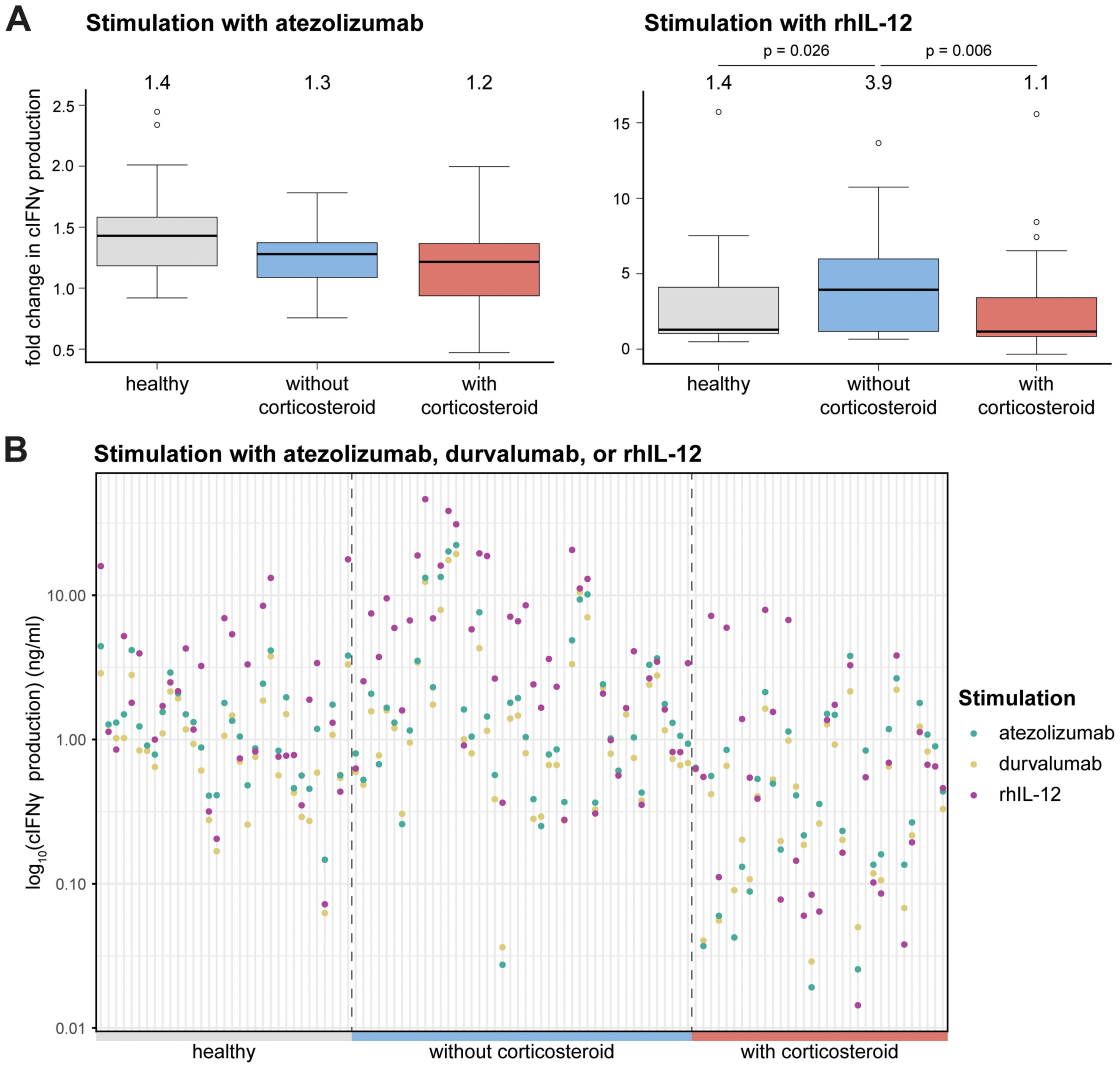
Immunomodulatory effects on cIFNγ production. **(A)** Fold change compared to SEB stimulated in cIFNγ production upon stimulation with atezolizumab (left) and rhIL-12 (right). Each graph displays boxplots for different groups on the x-axis: healthy, tumor-bearing dogs without corticosteroid treatment and tumor-bearing dogs with corticosteroid treatment and the fold change in cIFNγ production on the y-axis. The boxplots show median, 1^st^ and 3^rd^ quantile, with the median value shown above each. Mann-Whitney U test was performed for statistical analysis. Only p-values below 0.05 are shown. **(B)** Log_10_ transformed cIFNγ production (ng/ml) among different groups (healthy, tumor-bearing dogs without corticosteroid treatment and tumor-bearing dogs with corticosteroid treatment) after stimulation with atezolizumab, durvalumab or rhIL-12.

To evaluate how prior corticosteroid treatment affects canine lymphocyte responsiveness to PD-1/PD-L1 axis blockade or rhIL-12, we assessed the ex vivo responses of cancer patient-derived PBMCs (**Supplementary Figure 1**). Stimulation of cPBMCs with SEB, a polyclonal activator, induces activation, IFNγ production, and subsequent PD-1/PD-L1 upregulation. Blockade of the PD-1/PD-L1 axis usually leads to a moderate further increase in cIFNγ levels after 72 hours in healthy donor PBMCs (23). Atezolizumab stimulation in healthy and tumor-bearing dogs, with or without corticosteroid treatment, showed comparable medians for all groups but with slightly lower levels of cIFNγ production in tumor-bearing dogs and even more so corticosteroid treated tumor-bearing dogs (**Figure 2A**). In contrast, rhIL-12 treatment directly induced higher cIFNγ levels compared to PD-1/PD-L1 axis blockade, though with higher variability (**Figure 2A**). Significant differences in the relative cIFNγ increase were observed for stimulation with rhIL-12 between tumor-bearing corticosteroid treated and non-treated groups and between healthy and tumor-bearing non-treated groups, but not between healthy and tumor-bearing corticosteroid treated group (**Figure 2A**). Individual analysis showed rhIL-12 elicited markedly higher fold-change of cIFNγ levels than atezolizumab (**Figure 2B**). As expected, durvalumab, used here as a human IgG isotype control, produced low cIFNγ as it does not bind canine PD-L1 (23) (**Figure 2B**). Although no statistical comparison was possible on an individual level, tumor-bearing corticosteroid-treated dogs displayed visibly lower cIFNγ outputs after stimulation (**Figure 2B**). Of note, research-grade gilvetmab demonstrated comparable performance to atezolizumab and significantly increased cIFNγ production of healthy beagle cPBMCs in this assay (**Supplementary Figure 5**). Sample availability prevented a repetition of the study with patient-derived PBMCs. With its silenced Fc (34) atezolizumab does not trigger antibody-dependent, cellular cytotoxicity (ADCC) of PD-L1 positive cells, but prevents ligand binding to PD-1 (23). It is thus conceivable that our atezolizumab-based findings may also apply to gilvetmab-based inhibition of this signaling axis.

**Figure 3:**
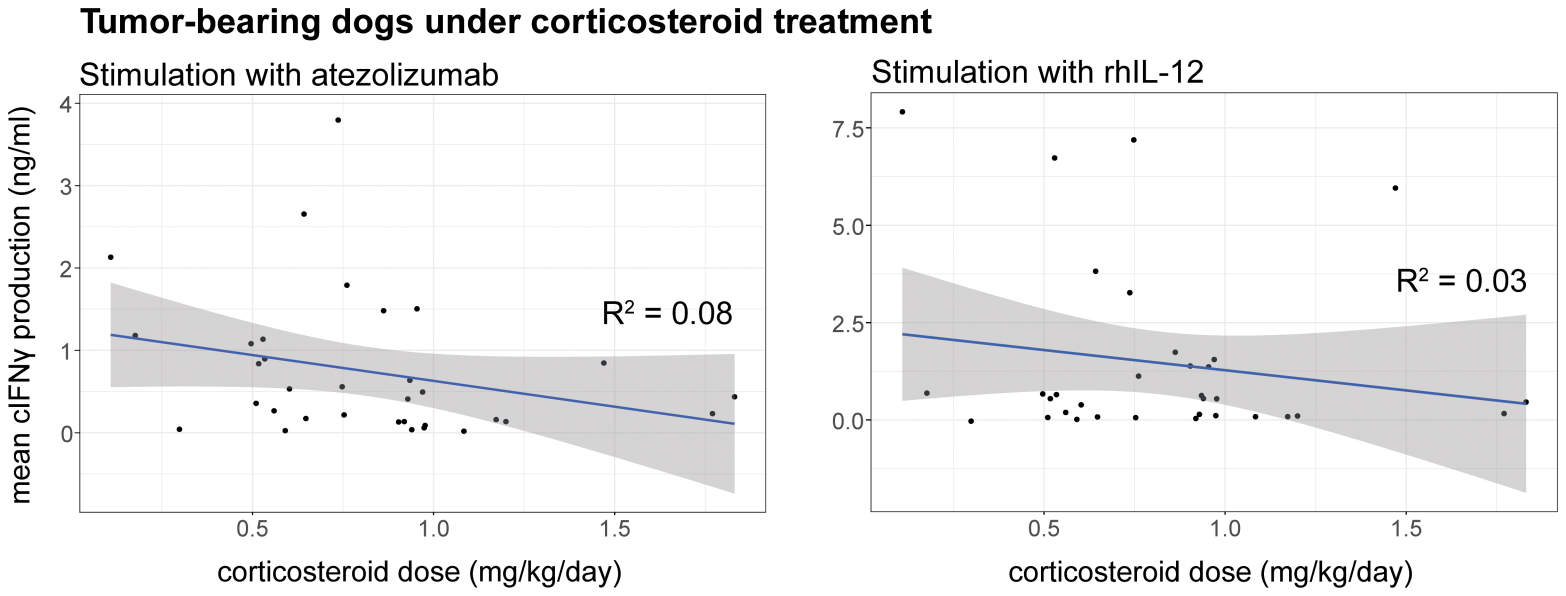
Correlation between cIFNγ production (ng/ml) and corticosteroid (mg/kg/day) dose upon stimulation of cPBMCs with atezolizumab (left) or rhIL-12 (right). Linear regression was applied to display a trendline together with the 95% confidence interval. R squared was calculated for each correlating trendline. p-values were calculated for rhIL-12 (p = 0.33) and atezolizumab (p = 0.11) for each linear regression.

To evaluate whether the dose of corticosteroid administration influences the response to treatment, cIFNγ levels were plotted against corticosteroid doses upon ex vivo stimulation with rhIL-12 or atezolizumab. No clear dose cut-off emerged; however, linear regression indicated a weak, non-significant trend towards decreased cIFNγ production at higher mg/kg/day corticosteroid dose (**Figure 3**). In addition, it is important to note that dogs receiving ≤1mg/kg corticosteroids showed variable cIFNγ production, with some producing almost none and others producing high levels.

Finally, we determined whether the administration of corticosteroids can be used as a predictive factor for cIFNγ production upon stimulation with rhIL-12 or atezolizumab. The performed logistic regression and ROC curve with all the complete cases showed significantly bigger areas under the ROC curve when corticosteroid administration was considered for the stimulation with rhIL-12 and atezolizumab (**Supplementary Figure 3**). To predict cIFNγ production of patients’ PBMCs stimulated with rhIL-12 or atezolizumab, corticosteroid treatment needs to be taken into consideration since the predicted outcome will be more accurate. This supports the above findings that corticosteroid administration does influence the response to treatment.

### Prior corticosteroid treatment affects the cPBMC composition

To contextualize ex vivo responses and potential shifts in peripheral lymphocyte populations, we performed a flow cytometric analysis on the cPBMCs from healthy and tumor-bearing dogs, comparing immune cell compositions across groups with and without corticosteroid treatment.

**Figure 4:**
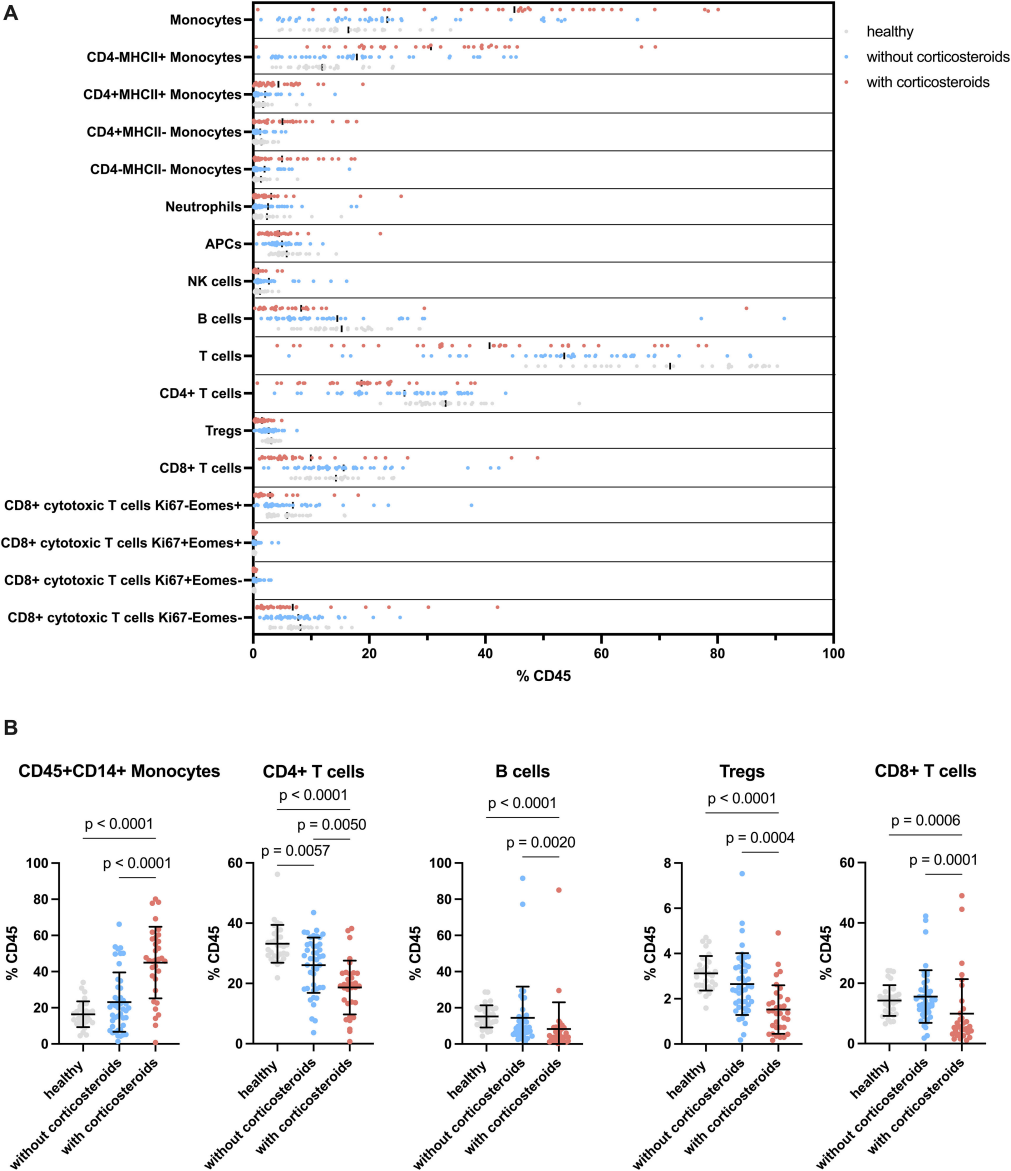
Flow cytometric analysis of unstimulated cPBMCs. **(A)** Distribution of cell populations separated by groups. Showing the different cell types as a percentage of CD45+ cells divided by healthy, tumor-bearing without corticosteroid treated and tumor-bearing with corticosteroid treated dogs. **(B)** Selected cell types displayed separated by group on the x-axis and percentage of CD45+ cells on the y-axis. The groups are compared for significant changes in cell composition. Each dot indicates an individual dog. The graphs show mean ± SD. One-way ANOVA followed by Kruskal-Wallis test was applied for statistical analysis. Only p values below 0.05 are shown.

Notable alterations were observed in CD45+ blood leukocytes among healthy dogs, non-treated tumor-bearing dogs, and corticosteroid-treated tumor-bearing dogs (**Figure 4A**). Monocytes (CD45+CD14+) showed no significant difference between healthy and non-treated tumor-bearing dogs but were significantly increased in corticosteroid-treated tumor-bearing dogs compared to both healthy and non-treated tumor-bearing dogs (**Figure 4B**). This increase affected all CD4 and MHCII defined monocyte subsets (**Supplementary Figure 4A**). In contrast, lymphoid populations displayed opposite trends. CD4+ T cells decreased in non-treated tumor-bearing dogs compared to healthy dogs, with a further significant reduction in corticosteroid-treated tumor-bearing dogs (**Figure 4B**). Similarly, B and regulatory T cells (Tregs) showed moderate yet significant decreases in corticosteroid-treated tumor-bearing dogs (**Figure 4B**). NK cells increased modestly in non-treated tumor-bearing dogs compared to healthy dogs but then decreased significantly in corticosteroid-treated tumor-bearing dogs relative to non-treated tumor-bearing dogs (**Supplementary Figure 4B**). CD8+ T cells significantly decreased in corticosteroid-treated tumor-bearing dogs compared to both groups (**Figure 4B**). Among these, Eomes+ CD8+ T cells were significantly reduced in corticosteroid-treated tumor-bearing dogs, as were the few Ki67+ Eomes+ and Ki67+ Eomes- CD8 T cell populations (**Supplementary Figure 4C**). In summary, while the overall distribution of cell counts varied, significant differences were found in numerous subsets which might explain the variability in response to stimulation.

## Discussion

Studies demonstrated that blocking the canine PD-1/PD-L1 axis enhances cIFNγ production and reactivates exhausted lymphocytes in dogs (35), similar to human settings (36). In veterinary medicine, immune checkpoint inhibitor therapy is primarily available in preclinical or trial settings, apart from gilvetmab, a recently approved canine PD-1 inhibitor. Introduced in 2023, gilvetmab’s approval is limited to the US and Canada and specific tumor types, such as mast cell tumors and melanomas (22). In addition, no studies have yet examined the impact of corticosteroid administration on responses to ICIs or IL-12 treatments in dogs. In human medicine, corticosteroids are known immunosuppressors often associated with reduced ICI efficacy (37, 38), yet reliable biomarkers for predicting treatment responses remain elusive (39). Similarly, in veterinary medicine, corticosteroids impair the immune system in a dose-dependent manner, affecting complement function and antibody responses (19, 40). This study analyzed the immunological effects of corticosteroid pretreatment on cPBMCs, focusing on the induced changes in the PBMC composition and their ex vivo response to PD-1/PD-L1 ICIs or IL-12 cytokine therapy.

Our findings reveal significant changes in immune cell populations among tumor-bearing dogs, especially with corticosteroid treatment. While the monocyte population (CD45+CD14+) remained unchanged between healthy and non-treated tumor-bearing dogs, corticosteroid treatment markedly increased this population, consistent with its role in enhancing monocyte recruitment or survival (41). Corticosteroid-treated dogs exhibited increased CD4-MHCII+ monocytes, which may impair antigen presentation and T-cell activation (42, 43). Corticosteroids are known to downregulate MHCII expression on monocytes and dendritic cells, disrupting their antigen-presenting capacity (43), and their association with regulatory monocytes in humans potentially supports their immunosuppressive role in this study (42). Corticosteroid treatment also decreased B cells, CD4+ effector and Tregs, and CD8+ T cells, aligning with known effects, such as T cell apoptosis and diminished proliferation (44–46). The decline in CD4+ T cells in non-treated tumor-bearing dogs, further exacerbated by corticosteroid treatment, suggests impaired adaptive immunity, that could weaken antitumor responses (44). Moreover, corticosteroid-induced reductions in Ki67- Eomes+ memory CD8+ T cells point to compromised long-term immune memory against tumor antigens, increasing the risk of tumor recurrence (47). This mirrors human studies where corticosteroids impair CD8+ T cell cytotoxicity, adversely affecting tumor control (48). Similarly, reductions in activated Ki67+ CD8+ T cells were observed, underscoring corticosteroids’ broad suppressive effects on adaptive and innate immunity (49, 50). Healthy dogs showed low NK cell frequencies while tumor-bearing dogs exhibited significant increases, consistent with NK cells’ role in cancer immunosurveillance (51). However, corticosteroid-treated dogs showed a sharp decline in NK cells, suggesting impaired immune surveillance critical for tumor control (52). Corticosteroid pretreatment also impaired the activation of PBMCs, reflected by reduced IFNγ output following stimulation, likely due to the reduction of NK and T cells (53) which are primary contributors to IFNγ production.

We further assessed the impact of corticosteroid pretreatment on responses to PD-L1/PD-1 axis blockade and rhIL-12. While no significant differences in IFNγ production were found between corticosteroid-treated and non-treated groups, the variability in individual responses was substantial. Trends indicated that higher corticosteroid doses and longer treatment durations correlated with lower IFNγ output, highlighting dose-dependent immunosuppressive effects (19). Although higher doses negatively influenced IFNγ production, these effects were not statistically significant at median values, suggesting that the corticosteroid doses in this study remain acceptable for ICI therapy. Importantly, they indicate that corticosteroid pretreatment should not preclude ICI administration, reflecting similar findings in human medicine (18, 20). This variability, consistent with findings in human oncology, underscores the challenges of predicting immune responses and tailoring therapies (18, 20). Cytokine production and flow cytometry analysis of healthy human PBMCs upon ICI stimulation proposed, among others, IFNγ as a good predictive marker for treatment response even though a high variability in production was detected as well (54). Although our study only reports on ex vivo testing, studies in human medicine suggest that the composition of PBMCs derived from patients before and during ICI treatment is predictive of treatment response (55, 56). In addition, IFNγ ex vivo production may serve as a biomarker for overall survival time in lung cancer patients (57) further supporting extrapolation of the results of this ex vivo study to patients.

With regards to the independence of individual samples, eight dogs were sampled twice. Seven of these were healthy blood donors and regularly screened for a variety of conditions, including a complete hematology, where any irregularities in white blood cell count would have led to the exclusion of the aforementioned donors. The inclusion of non-independent samples by taking multiple samples from a patient poses a limitation. While efforts were made to account for this, such as taking repeated samples at least six months apart from each other, future studies should consider study designs that ensure complete independence of observations.

No randomization for corticosteroid administration took place in this study, since the treatment was conducted per clinician’s choice. Brain tumor-bearing dogs received corticosteroids more frequently than any other tumor type, and more tumor-bearing dogs were included than healthy controls, reflecting real-world variability. Moreover, it is well established that tumors influence the immune system (58), and intracranial tumors can trigger systemic lymphopenia in humans (59, 60). We can therefore not exclude that differences in biology between tumor location, stage and the effect of intra- and extracranial tumors influence the peripheral immune cell composition and ultimately also the response to ICIs. Tumor-induced changes in leukocyte composition likely contributed to broader data distributions within the tumor-bearing group (61, 62) and warrant further investigations of individual cancer types and stages.

In summary, the above challenges certainly impose some limitations on our findings, but at the same time need to be taken into account for future study designs due to their alignment with the current routine veterinary practices. Future studies could standardize corticosteroid dosing and duration to minimize confounding factors and improve therapeutic predictability. In addition, longitudinal sampling can help to identify the duration of the influence of corticosteroids and would allow the determination of an optimal treatment timepoint for ICI and/or cytokine therapy. Bearing in mind the limitations of PBMC ex vivo responses upon polyclonal stimulation to predict anti-cancer immune responses *in vivo*, results from this heterogeneous population of canine cancer patients suggest that corticosteroid pretreatment at the doses and durations used should not exclude dogs from ICI therapy. While our findings demonstrate the potential for translating human oncology approaches (18, 20) to veterinary medicine, they underscore the importance of harmonizing immunotherapeutic strategies with existing therapeutic regimens. With the approval of gilvetmab, which reacted comparably to atezolizumab in our experiments, further clinical testing and routine use of ICI in canine cancer patients is warranted, and corticosteroid dosage and duration are likely important factors influencing treatment outcome.

## Data Availability

The raw data supporting the conclusions of this article will be made available by the authors, without undue reservation.
