# Supplementary material for: Prior corticosteroid treatment alters cPBMC composition and IFNγ response to immunotherapy in canine cancer
Source: Front Immunol. 2025 Apr 24;16:1544949. doi: 10.3389/fimmu.2025.1544949 (PMC12058859; doi:10.3389/fimmu.2025.1544949)
Supplement: Supplementary file 1 [file DataSheet1.pdf]

Supplementary Material

Prior corticosteroid treatment alters cPBMC composition and IFNɣ response to immunotherapy in canine cancer

Anna Barbara Emilia Zimmermann[1,2], Betül Taskoparan[2], Daniel Fuchs[1,3], Stanislav Pantelyushin[2], Mathischan Maheswaran[2], Manuela Schnyder[4], Sonja Hartnack[5], Carla Rohrer Bley[1], Johannes vom Berg[2*]

[1]Clinic for Radiation Oncology & Medical Oncology, University Animal Hospital, Vetsuisse Faculty, University of Zurich, Zurich, Switzerland
[2]Institute of Laboratory Animal Science, Vetsuisse Faculty, University of Zurich, Schlieren, Switzerland
[3]Institute of Veterinary Pharmacology and Toxicology, Vetsuisse Faculty, University of Zurich, Zurich, Switzerland
[4]Institute of Parasitology, Vetsuisse Faculty, University of Zurich, Zurich, Switzerland
[5]Section of Epidemiology, Vetsuisse Faculty, University of Zurich, Zurich, Switzerland

*** Correspondence:**
johannes.vomberg@uzh.ch

Supplementary Figure 1-5
Supplementary Table 1-3

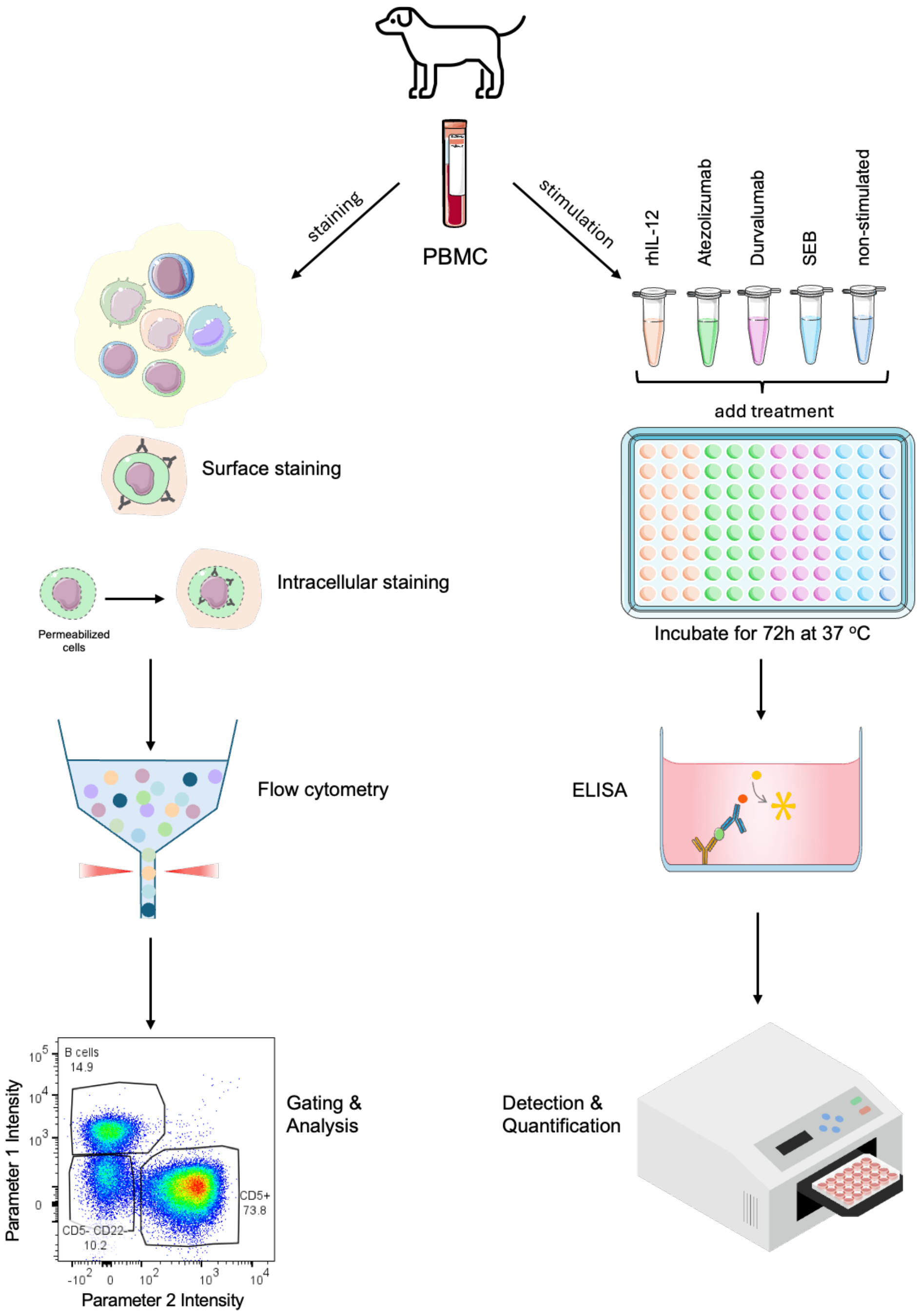

Supplementary Figure 1. Graphical abstract. After collecting blood samples from dogs enrolled in the study, cPBMCs were collected and stored at -80°C until usage for either flow cytometry analysis or interferon-gamma ELISA. For flow cytometry analysis (left), cPBMCs were stained using antibodies for the surface staining followed by antibodies for the intracellular staining after permeabilization of the cells. Sorting was performed by BD LSR Fortessa II and analysis was conducted with FlowJo software. For ELISA analysis (right), cPBMCs were cultivated either unstimulated or stimulated with staphylococcal enterotoxin B (SEB) as a single agent or in combination with atezolizumab, durvalumab or rhIL-12 for 72 hours at 37°C. Afterwards, ELISA was performed with the supernatant and the absorbance levels were measured using SPARKS TECAN.

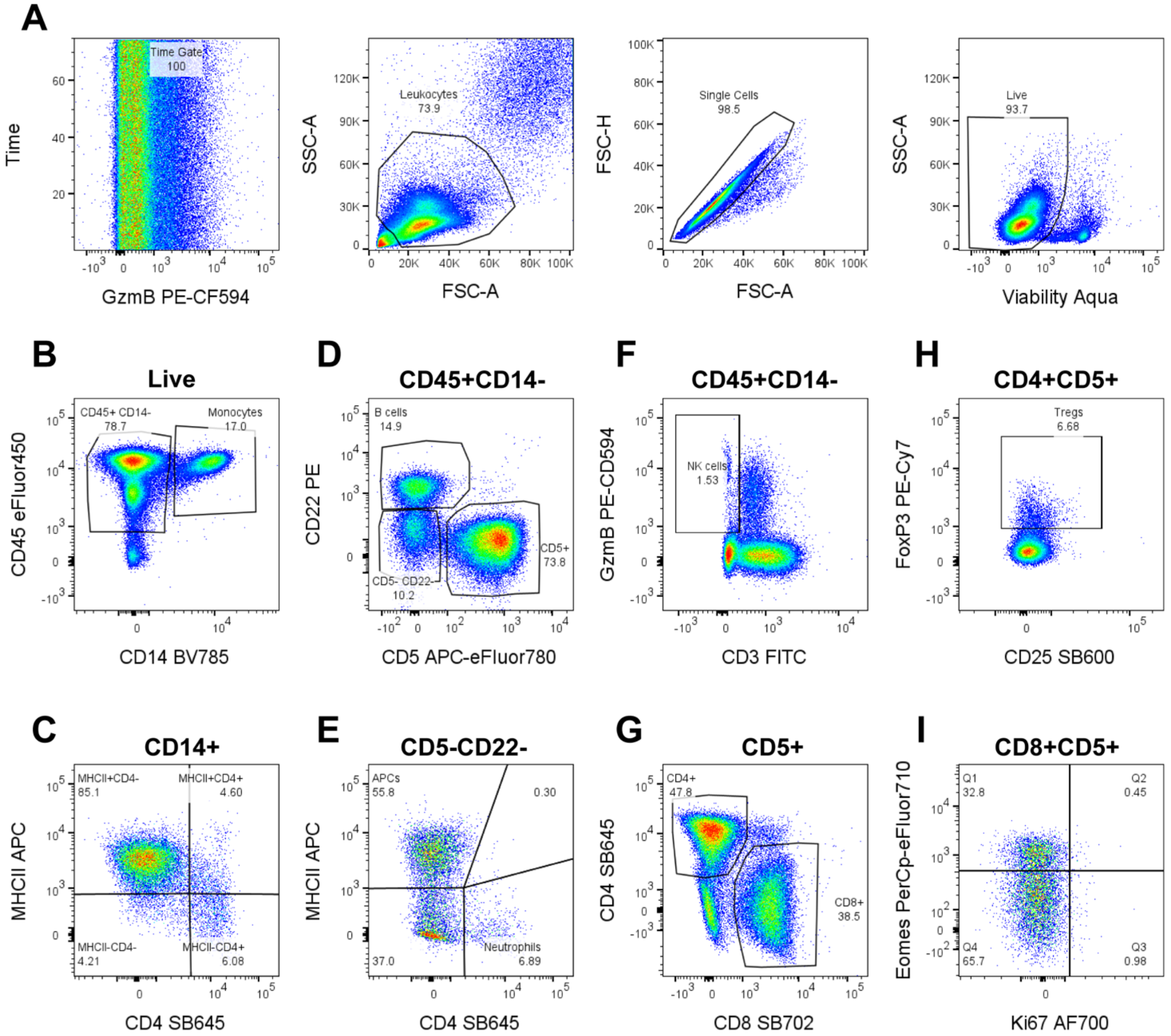

Supplementary Figure 2. Exemplary gating strategy on cryopreserved canine PBMCs obtained from dogs presented at the Small Animal Clinic of Veterinary Medicine Zurich. Ficoll density gradient centrifugation from whole canine blood stained and subsequently acquired on a BD LSR Fortessa II as previously described. **(A)** Gating for live single cells, excluding debris and doublets. **(B)** Gating for monocytes (CD14+) and other leukocytes (CD45+CD14−). **(C)** MHCII and CD4 expression on CD14+ monocytes. **(D)** Gating strategy for lymphocyte lineages (CD22+ B cells and CD5+ T cells). **(E)** MHCII and CD4 expression on CD22-CD5- cells, which are separated into MHCII+ antigen presenting cells (APCs) and CD4+MHCII- neutrophils. **(F)** Identification of putative NK cells by expression of GranzymeB+ CD3−. **(G)** Gating for T cell subsets using CD8 and CD4. **(H)** Identification of regulatory T cells (Tregs) and their expression of CD25. **(I)** Transcription factor Eomes and proliferation marker Ki67 expression on CD8+ T cells. Parent gates are indicated above of the plots in bold where necessary.

Breed	Healthy patients (sampled)<br>n (*multiple samples)	Tumor patients (sampled)<br>n (*multiple samples)
Akita	0 (0.0%)	1 (1.3%)
Alano	1 (4.2%)	0 (0.0%)
Alaskan Malamute	0 (0.0%)	1 (1.3%)
American Bulldog	0 (0.0%)	2 (2.6%)
Australian Shepherd	0 (0.0%)	2 (2.6%)
Barsoi	1 (4.2%)	0 (0.0%)
Beagle	0 (0.0%)	1 (1.3%)
Bauceron	1 (4.2%)	0 (0.0%)
Belgian Shepherd Dog	0 (0.0%)	1 (1.3%)
Berger Blanc Suisse	1 (*2) (4.2%)	0 (0.0%)
Bernese Mountain Dog	3 (12.5%)	2 (2.6%)
Bobtail	2 (8.3%)	0 (0.0%)
Border Collie	0 (0.0%)	2 (2.6%)
Boston Terrier	0 (0.0%)	2 (2.6%)
Boxer	1 (*2) (4.2%)	7 (9.2%)
Collie	0 (0.0%)	1 (1.3%)
Continental Bulldog	1 (*2) (4.2%)	0 (0.0%)
Crossbreed	1 (*2) (4.2%)	18 (23.7%)
Czechoslovakian Wolfdog	0 (0.0%)	1 (1.3%)
Flat Coated Retriever	1 (4.2%)	2 (2.6%)
French Bulldog	0 (0.0%)	9 (11.8%)
Bavarian Mountain Hound	0 (0.0%)	1 (*2) (1.3%)
German Shepherd	1 (4.2%)	1 (1.3%)
Golden Retriever	3 (*4) (12.5%)	6 (7.9%)
Greyhound	1 (*2) (4.2%)	0 (0.0%)
Groenendael	0 (0.0%)	1 (1.3%)
Standard Poodle	0 (0.0%)	1 (1.3%)
Hungarian Viszla	0 (0.0%)	1 (1.3%)
Irish Soft Coated Terrier	0 (0.0%)	1 (1.3%)
Labradoodle	1 (*2) (4.2%)	0 (0.0%)
Labrador Retriever	1 (4.2%)	3 (4.0%)
Lagotto Romagnola	0 (0.0%)	1 (1.3%)
Pug	0 (0.0%)	1 (1.3%)
Miniature Poodle	1 (4.2%)	1 (1.3%)
Rhodesian Ridgeback	0 (0.0%)	4 (5.3%)
Giant Schnauzer	1 (4.2%)	0 (0.0%)
Swiss Mountain Dog	1 (4.2%)	0 (0.0%)
Segugio Maremmano	0 (0.0%)	1 (1.3%)
Hungarian Pointer	1 (4.2%)	1 (1.3%)
**Total**	**24**	**76**

Supplementary Table 1. Sampled dogs separated by tumor-bearing and healthy donors and listed per breed. (*) multiple samples taken from the same patient.

	Healthy	Tumor-bearing
	Mean [range min-max]	Mean [range min-max]
**Weight**	32.2kg [24.5-52.3kg]	23.8kg [6.55-52kg]
**Age**	4.8years [1-9years]	8.7years [3-14years]

Supplementary Table 2. Mean and range for age and weight of sampled dogs separated by healthy and tumor-bearing.

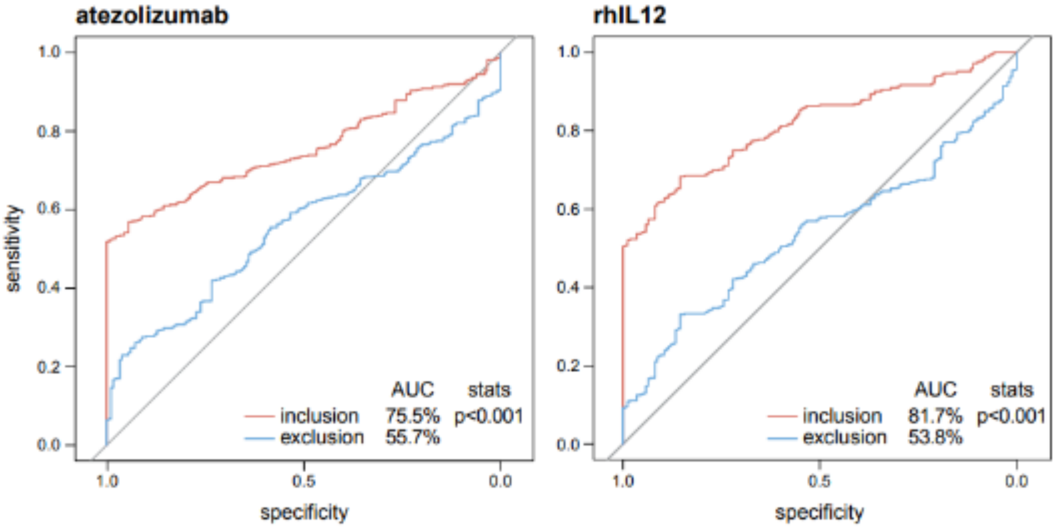

Supplementary Figure 3. ROC curve for determination whether inclusion of corticosteroid administration influences the ability to distinguish between tumor-bearing or healthy dogs with regards to cIFNγ production after stimulation. Logistic regression analysis for evaluation of the predictive value of atezolizumab (left) or rhIL-12 (right) with (red) and without (blue) the inclusion of corticosteroid administration distinguishing between tumor-bearing and healthy dogs.

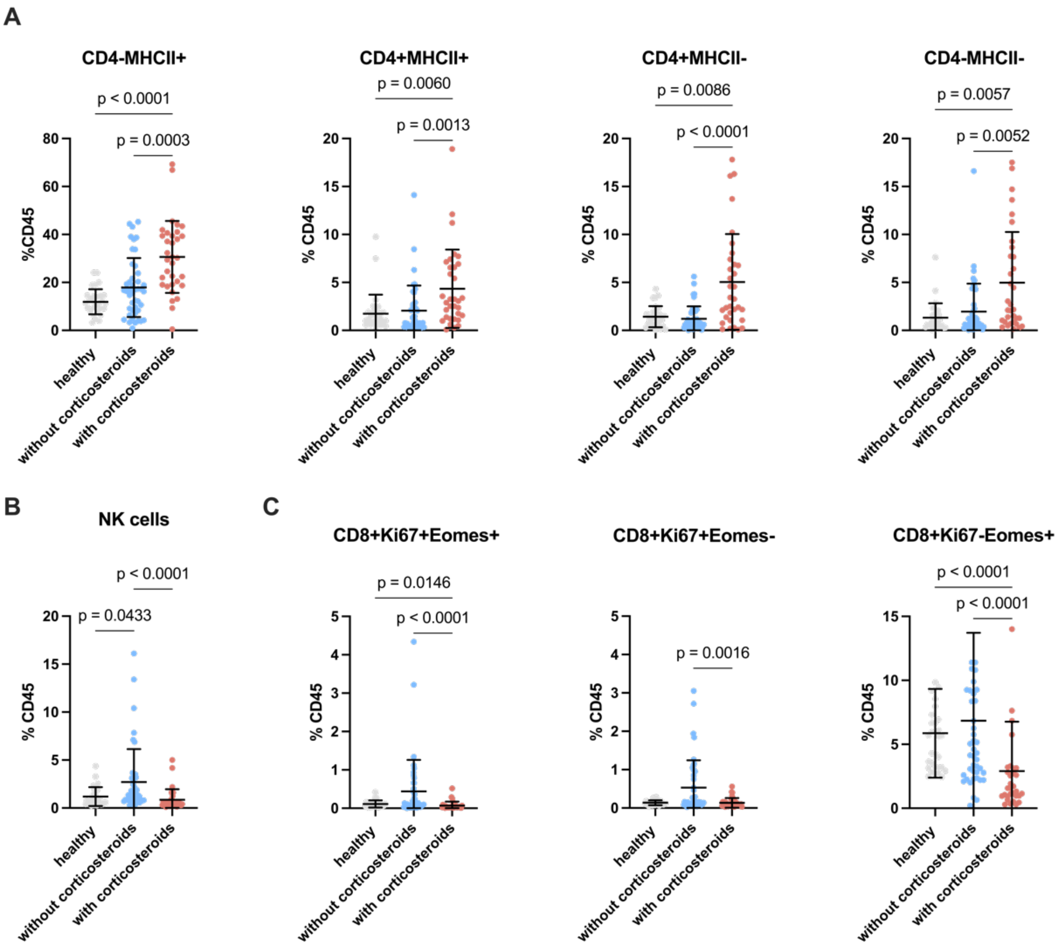

Supplementary Figure 4. Flow cytometry analysis of unstimulated cPBMCs. **(A-C)** Selected cell types displayed separated by group on the x-axis and percentage of CD45+ cells on the y-axis. The groups are compared for significant changes in cell composition. Each dot indicates an individual dog. The graphs show mean ± SD. One-way ANOVA followed by Kruskal-Wallis test was applied for statistical analysis. Only p values below 0.05 are shown.

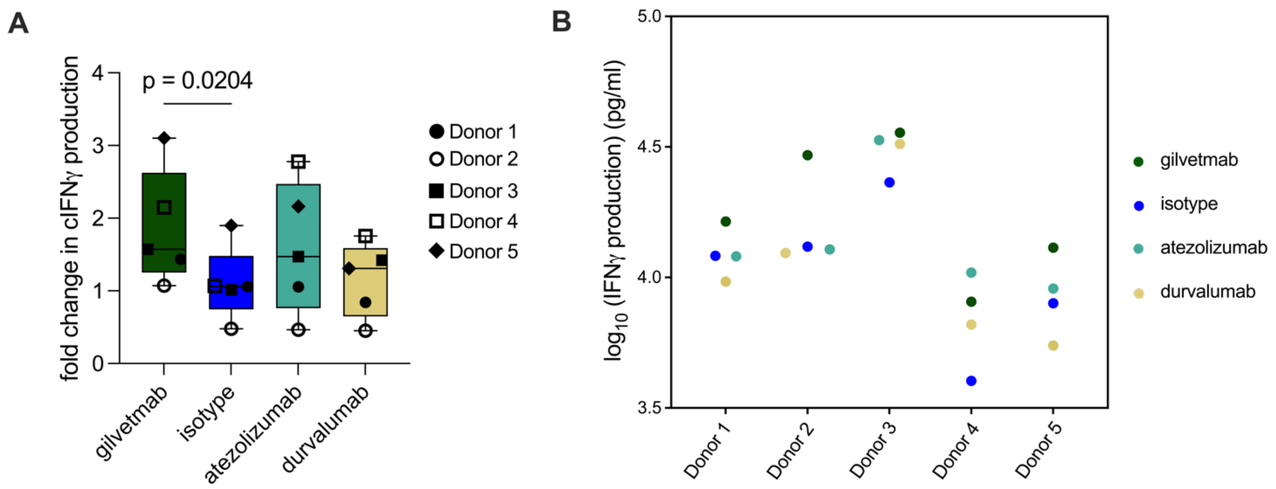

Supplementary Figure 5. Immunomodulatory effects of gilvetmab on healthy beagles. (**A)** Fold change cIFNγ production upon stimulation with gilvetmab, isotype control (canine IgG2), atezolizumab and durvalumab. Each icon represents an individual donor on the graph. The boxplots show mean. One-way ANOVA was performed for statistical analysis. Only p values below 0.05 are shown. **(B)** Log_{10} transformed cIFNγ production (pg/ml) among different treatments (gilvetmab, isotype control (canine IgG2), atezolizumab and durvalumab).

**Donor**	**Sex**	**Age (years)**	**Breed**
**1**	female intact	2	Beagle
**2**	male intact	2	Beagle
**3**	female intact	2	Beagle
**4**	male intact	2	Beagle
**5**	male intact	2	Beagle

Supplementary Table 3. Characteristics of healthy PBMC donors for the testing of gilvetmab.
